# Immunotherapy and radiotherapy for older patients with invasive bladder cancer unfit for surgery or chemotherapy: practical proposal by the international geriatric radiotherapy group

**DOI:** 10.3389/fonc.2024.1371752

**Published:** 2024-07-04

**Authors:** Nam Phong Nguyen, Ulf Lennart Karlsson, Brandi R. Page, Monica-Emilia Chirila, Vincent Vinh-Hung, Olena Gorobets, Meritxell Arenas, Mohammad Mohammadianpanah, Seyed Alireza Javadinia, Huan Giap, Lyndon Kim, Fabien Dutheil, Vedang Murthy, Abba Aji Mallum, Ghassen Tlili, Zineb Dahbi, Gokoulakrichenane Loganadane, Sergio Calleja Blanco, Satya Bose, Elena Natoli, Eric Li, Alessio G. Morganti

**Affiliations:** ^1^ Department of Radiation Oncology, Howard University, Washington, DC, United States; ^2^ Department of Radiation Oncology, International Geriatric Radiotherapy Group, Washington, DC, United States; ^3^ Department of Radiation Oncology, Johns Hopkins University, Baltimore, MD, United States; ^4^ Department of Clinical Development, MVision AI, Helsinki, Finland; ^5^ Department of Radiation Oncology, Amethyst Radiotherapy Centre, Cluj-Napoca, Romania; ^6^ Department of Radiation Oncology, Centre Hospitalier Public du Contentin, Cherbour-en-Contentin, France; ^7^ Department of Oral Surgery, University Hospital of Martinique, Fort-de-France, France; ^8^ Department of Radiation Oncology, Sant Joan de Reus University Hospital, University of Rovira, I Virgili, Tarragona, Spain; ^9^ Colorectal Research Center, Department of Radiation Oncology, Shiraz University of Medical Sciences, Shiraz, Iran; ^10^ Non-Communicable Diseases Research Center, Sabzevar University of Medical Sciences, Sabzevar, Iran; ^11^ Department of Radiation Oncology, Medical University of South Carolina, Charleston, SC, United States; ^12^ Division of Neuro-Oncology, Mount Sinai Hospital, New York, NY, United States; ^13^ Department of Radiation Oncology, Clinique Sainte Clotilde, Saint Denis, Reunion, France; ^14^ Tata Memorial Centre, Homi Bhabha National Institute, Mumbai, India; ^15^ Department of Radiation Oncology, University of KwaZulu Natal, Durban, South Africa; ^16^ Department of Urology, University Hospital Center, Sousse, Tunisia; ^17^ Department of Radiation Oncology, Mohammed VI University of Health Sciences, Casablanca, Morocco; ^18^ Department of Radiation Oncology, Institut Curie, Paris, France; ^19^ Department of Oral Maxillofacial Surgery, Howard University, Washington, DC, United States; ^20^ Department of Radiation Oncology, Istituto di Ricovero e Cura a Carattere Scientifico (IRCCS) Azienda Ospedaliero-Universitaria di Bologna, Bologna, Italy; ^21^ Radiation Oncology, DIMEC, Bologna University, Bologna, Italy; ^22^ Department of Pathology, Howard University, Washington, DC, United States

**Keywords:** older, bladder cancer, invasive, ICI, radiotherapy

## Abstract

The standard of care for non-metastatic muscle invasive bladder cancer is either radical cystectomy or bladder preservation therapy, which consists of maximal transurethral bladder resection of the tumor followed by concurrent chemoradiation with a cisplatin-based regimen. However, for older cancer patients who are too frail for surgical resection or have decreased renal function, radiotherapy alone may offer palliation. Recently, immunotherapy with immune checkpoint inhibitors (ICI) has emerged as a promising treatment when combined with radiotherapy due to the synergy of those two modalities. Transitional carcinoma of the bladder is traditionally a model for immunotherapy with an excellent response to Bacille Calmette-Guerin (BCG) in early disease stages, and with avelumab and atezolizumab for metastatic disease. Thus, we propose an algorithm combining immunotherapy and radiotherapy for older patients with locally advanced muscle-invasive bladder cancer who are not candidates for cisplatin-based chemotherapy and surgery.

## Introduction

Bladder cancer prevalence increases significantly with age. Old age is also associated with a high risk of death, likely due to pre-existing comorbidities (1). Currently, the standard approach for eligible patients with non-metastatic muscle-invasive bladder cancer (MIBC) consists of neoadjuvant chemotherapy followed by radical cystectomy, pelvic lymph node dissection, and urinary neobladder reconstruction. In radical cystectomy, genitourinary organs including the bladder, prostate, and seminal vesicle in male patients, and the bladder, uterus, ovaries, fallopian tubes, and anterior vaginal wall in female patients, should be resected (2). Radical cystectomy is a highly morbid surgical procedure that significantly compromises patients’ quality of life. In addition, less than half of older patients with MIBC receive definitive therapy either with surgical resection or transurethral resection of a bladder tumor (TURBT) followed by chemoradiation (3). Among patients with invasive bladder cancer who underwent radical cystectomy. the mortality rate was significantly higher in older patients one year after the procedure (4). Frailty prior to surgical resection is often prognostic of a high mortality rate after treatment (5). Thus, older and frail patients are not ideal candidates for surgery. Bladder preservation therapy with chemotherapy following maximal TURBT and radiation is often offered as an alternative for those patients.

A dose-dense MVAC (methotrexate, vinblastine, doxorubicin, and cisplatin) regimen is the most effective combined chemotherapy in urothelial carcinoma; however, due to its toxicity, only a minority of patients can tolerate this protocol (6). An alternative less toxic cisplatin-based chemotherapy regimen frequently used is gemcitabine and cisplatin (GC). For patients who are not candidates for cisplatin due to reduced kidney function, mitomycin and 5-fluorouracil (5-FU) may be offered as another alternative, even though this regimen may be more toxic (7). The National Comprehensive Cancer Network (NCCN) guidelines also recommend cisplatin alone, low-dose gemcitabine, or 5-FU and mitomycin as preferred radiation sensitizers. However, older patients are infrequent candidates for cisplatin-based chemotherapy due to the high prevalence of chronic renal failure (CRF). It is estimated that 39.4% of Americans over the age of 60 would develop CRF (8). Those who are 75 years or older are at higher risk of end-stage renal failure requiring dialysis (9). Frailty, which significantly increases with age, is also associated with an adverse outcome following chemotherapy or chemoradiation (10, 11). Thus, older and frail patients with MIBC pose a treatment challenge to clinicians, as radiotherapy alone is less effective compared to concurrent chemoradiation for local control and survival (12).

Recent advances in immunotherapy have shed some light on how immune checkpoint inhibitors (ICI) may confer a survival advantage when combined with radiotherapy for MIBC due the synergy between those two modalities (13). Immunotherapy has been reported to be effective for local control in patients with non-muscle-invasive bladder cancer unresponsive to Bacille Calmette-Guerin (BCG) vaccine (14, 15). In addition, for locally advanced bladder cancer, neoadjuvant immunotherapy has been reported to induce a high rate of pathological response, and potentially improve survival through the reduction of occult distant metastases (16). Even though the data is still preliminary, it suggests that combining immunotherapy with a local therapy which may be synergistic with immunotherapy such as radiotherapy may further improve the response rate and increase the rate of anatomic bladder preservation (17).

The International Geriatric Radiotherapy Group (http://www.igrg.org) is an organization devoted to the care of older cancer patients, minorities, and women who are frequently excluded from clinical trials. Based on currently published literature, members of the genitourinary cancers subgroup propose in this article a practical protocol for older patients with MIBC who are too frail to undergo surgery or who are not candidates for chemotherapy (17). Radiotherapy and immunotherapy may induce long-term remission and potential cure for those patients.

## Rationale for using immunotherapy in bladder cancer

### Bladder cancer immune environment

Among many anatomic tumor types, bladder cancer has a unique tumor microenvironment which make it an ideal target for ICI.

Tumor mutation burden is a quantitative genomic biomarker that measures the number of mutations within a tumor (18). Higher expression of neoantigens by tumor cells leads to an increased accumulation of tumor-infiltrating lymphocytes (TIL) in the tumor microenvironment. These infiltrating lymphocytes come from the blood stream (B cells, T cells, natural killer cells, macrophages, dendritic cells etc. in various proportions) and adhere to tumor cells to kill them. There is a positive correlation between high TMB expression and TIL. TMB is measured by mutations per megabase of the cancel cell genomic (mut/Mb). Cancer cells that express 10mut/Mb or more are defined to have a high TMB (TMB-H). Among patients with bladder cancers, TMB-H tumor is associated with a better survival and disease-free survival (18–21). It is postulated that a high concentration of CD8 T cells, CD4 memory T cells, and NK cells in the tumor produces a better immune response (19). The correlation between high TIL in the tumor microenvironment and survival was corroborated in another study (22). A combination of TMB-H and a high concentration of TIL, defined as immune cell infiltration (ICI-H) in the tumor, provides the best prognosis for patients with MIBC (23). In addition, the frequency of mutations in mismatch repair (MMR) genes producing microsatellite instability is also significantly higher in TMB-H tumor, leading to a better response to immunotherapy (20).

Correlation between TMB-H and good prognosis for MIBC has been corroborated by other MIBC studies as those tumors are likely to respond to immunotherapy with ICI resulting in longer survival compared the ones with a low TMB (TMB-L) (24–26). A metaanalysis of 6,131 cancer patients treated with ICI reported a significant improvement in survival and progression-free survival for those with TMB-H (25). However, a higher cutoff value of 20 mut/Mb or more was correlated with a better survival, as it was a compilation of many cancers with different anatomic sites and different tumor microenvironments. For cancers with traditionally high TMB such as melanoma, colorectal, bladder, and non-small cell lung cancer, a cutoff value of 13 mut/Mb was reported (26). Thus, TMB value should be incorporated in any prospective study for MIBC.

In addition to TMB, program death ligand-1 (PD-L1) is another biomarker which has been reported to be associated with a poor prognosis and a better immune response to ICI in bladder cancer. Overexpression of PD-L1 by bladder tumor is frequently associated with a high tumor grade, poor response to BCG vaccine, stage progression and poor survival (27, 28). The role of PD-L1 is to help the tumor cells escape killing by the immune system. Binding to PD-L1 to the program cell dead-1 (PD-1) present on T cells leads to inhibition of their activation. The mechanism of T cell inhibition is complex and ranges from apoptosis to T cell exhaustion (29). An increase in PD-L1 expression has been reported in non-invasive bladder cancer after BCG treatment, suggesting that this biomarker confers resistance to intravesical bladder vaccination and subsequent disease progression (30). Depending on the cutoff value, the prevalence of PD-L1 ranges from 26% to 58% in bladder tumor specimens (31–33). High expression of PD-L1 is correlated with a poor response to chemotherapy (33). Radiotherapy significantly increases PD-L1 expression of bladder cancer cells in both *in vitro* and *in vivo* experiments, as the tumor produces an immunosuppressive environment through inhibition of CD-8 T cells to escape radiation killing (32). Conversely, high PD-L1 expression confers an excellent response to immunotherapy with ICI (34, 35). Thus, combining both TMB and PD-L1 expression may be advantageous to predict the response to immunotherapy for MIBC (36).

### Effectiveness of immunotherapy for bladder cancer

#### The role of ICI for non-muscle invasive bladder cancer

Radical cystectomy is the treatment of choice following BCG-unresponsive high grade non-muscle-invasive bladder cancer (NMIBC). However, many patients are unfit for surgery due to their age and co-existing morbidity. For those patients, a phase II study with atezolizumab every three weeks for one year has reported a biopsy-proven 26% complete response (CR) at six month (37). Treatment toxicity is acceptable, with 9 out of 73 patients (12.3%) developing grade 3–5 toxicity. One death was reported. Another report of 96 patients with NMIBC unresponsive to BCG also corroborated the efficacy and low toxicity of pembrolizumab (14). At a median follow-up of 36.4 months, 39 patients (41%) had CR. There was no treatment-related death. Eight patients (8%) developed grade 3–4 complications. Those two studies illustrated the proof of concept that ICI is effective for NMIBC *in vivo* due to the high PD-L1 expression of tumor cells (38).

#### The role of ICI for non-metastatic MIBC

Complete pathologic response (pCR) following neoadjuvant chemotherapy for bladder cancer is predictive of an excellent prognosis. Induction chemotherapy may decrease the rate of occult distant metastases and confer better survival for those patients. Indeed, a meta-analysis of 13 studies using neoadjuvant cisplatin-based chemotherapy reported excellent survival and relapse-free survival for patient who achieved pCR compared to those with residual disease in the surgical specimen (39). In addition, compared to patients undergoing radical cystectomy alone for invasive bladder cancer, induction chemotherapy has been reported to improve survival likely due to a reduction of distant metastases with systemic therapy (40).

Thus, investigations have been performed to assess whether neoadjuvant immunotherapy can achieve the same role as chemotherapy either for all chemotherapy naïve patients or for those who cannot receive cisplatin due to reduced kidney function. Immunotherapy with various ICI for two to three cycles before radical cystectomy was performed to assess pCR and survival for patients with locally advanced bladder cancer (41–50). The impact of biomarkers on response rate has also been investigated in selective studies.

Bandini et al. (41) reported 112 patients clinical stage T2-T4N0 who underwent neoadjuvant pembrolizumab for three cycles before radical cystectomy. The pCR rate was 37.5%. There was a positive correlation between TMB value and PD-L1 expression with pCR rate. However, on multivariate analysis, only PD-L1 expression was correlated with a high pCR rate. In a follow-up study of 155 patients, both TMB and PD-L1 have been reported to be associated with excellent event-free survival (EFS). The 3-year EFS was 87.3% and 89.8% for high TMB and PD-L1, respectively (43). Thus, the study highlighted the importance of those biomarkers to predict a good response to immunotherapy and survival. Correlation between high PD-L1 and TMB rate and high pCR rate was also reported after pembrolizumab among 34 patients with non-clear cell histology. The pCR was 37% (45).

Powles et al. (42) reported a 31% pCR following two cycles of atezolizumab and cystectomy for 95 patients with locally advanced bladder cancer. The pCR rate for PD-L1 positive patients was 37%. It was unclear what the PCR rate for PD-L1 negative patients was, but the difference did not achieve statistical significance. On the other hand, high CD8 level within the tumor was associated with a high pCR rate. The pCR rate was 40% and 20% for patients with high and low CD8 levels, respectively.

Nivolumab alone or in combination with another agent was investigated for neoadjuvant locally advanced bladder cancer in two studies. The pCR rate for nivolumab alone was 17% (47). It was unclear whether this lower pCR rate was attributed to the administration of the drug schedule, as patients only received two cycles before surgery. However, when combined with ipililumab with the same treatment schedule, there was a significant increase in the pCR rate. The PCR rate was 42.9% independent of CD8 level (48). The study suggests that combining immunotherapy with another biologic agent or another treatment modality such as radiotherapy may enhance the effectiveness of immunotherapy, leading to a better survival and potential bladder preservation.

Real-world data and other studies also support the use of neoadjuvant immunotherapy for bladder cancer. Using a propensity score matching method, Grassauer et al. (49) reported the survival and outcome of 840 patients who had surgery alone (n=280), neoadjuvant chemotherapy (n-=280), and neoadjuvant immunotherapy (n=280) for their locally advanced bladder cancer. The pCR rate was 26.4% and 22.5% for the neoadjuvant chemotherapy and immunotherapy, respectively. Survival rate was similar for both chemotherapy and immunotherapy and was significantly superior compared to the surgery-alone group. **Table 1** summarizes relevant neoadjuvant immunotherapy for bladder cancer.

**Table 1:** Neoajuvant immunotherapy for non-metastatic invasive bladder cancer.

Studies	Patient No	Immunotherapy	PCR	Biomarkers correlation
Bandini et al (41)	112	pembrolizumab 3 cycles	37.5%	PD-L1
Powles et al (42)	95	atezolizumab 2 cycles	31%	CD8+ expression
Basile et al (43)	155	pembrolizumab 3 cycles	36.8%	PD-L1, TMB>11.5
Hu et al (44)	48	tislelizumab	14.6%	NS
Necchi et al (45)	34	pembrolizumab 3 cycles	37%	PD-L1, TMB>11.5%
Li et al (46)	39	pembrolizumab 3 cycles	32.1%	NS
Grivas et al (47)	13	nivolumab 2 cycles	17%	NS
Van Dijk et al (48)	24	nivolumab+ipilumomab 2 cycles	46%	Independent
Grassauer et al (49)	280	NS	22.5%	NS

PCR, pathologic complete response; TMB, tumor mutation burden; NS, not specified.

Taken together, these studies suggest that neoadjuvant immunotherapy may be a viable option for patients who are not candidates for chemotherapy due to a high pCR rate and may also serve as a template for patients for desire anatomic bladder preservation, such as radiotherapy. Biomarkers such as PD-L1 and TMB should be included in any prospective studies for locally advanced MIBC, as they may be predictive of the response rate to ICI.

### The role of ICI for metastatic MIBC

The effectiveness of immunotherapy alone and standard chemotherapy has been tested in a randomized study for metastatic bladder carcinoma in the first-line setting. The median survival was 15.7 and 13.1 month, for atezolizumab and chemotherapy, respectively. However, serious adverse events resulting in withdrawal of the medication was significant less among patients who had ICI, at 6% and 34%, respectively (51). In a previous study, the response rate to atezolizumab was correlated with PD-L1 expression on tumor-infiltrating immune cells (52). In another study testing atezolizumab against chemotherapy in the second line setting for patients with metastatic bladder cancer refractory to platinum-based chemotherapy, there was no survival advantage for immunotherapy, but the response duration was longer and the adverse events were reduced compared to chemotherapy (52) Another anti-PD-L1 agent, durvalumab, did not improve survival in the first-line setting for metastatic bladder cancer patients. However, among patients with PD-L1 positive cancer, median survival was significantly longer compared to those receiving chemotherapy (53). These studies emphasized that selection of patients for immunotherapy was the key for its success.

Another PD-1 inhibitor, pembrolizumab, has been reported to improve survival compared to salvage chemotherapy among patients who relapsed following cisplatin-based chemotherapy. The 2-year survival was 26.9% and 14.3% for pembrolizumab and chemotherapy, respectively. Grade 3 or more side effects were also significant less with immunotherapy, at 16.5% and 50.2%, respectively (54). Pembrolizumab also conferred significant survival as first-line treatment for patients with locally advanced or metastatic urothelial cancer who were not eligible for cisplatin chemotherapy, especially among those with significant PD-L1 expression (55).

A comprehensive Cochrane systemic review and meta-analysis evaluated the effectiveness and safety of immunotherapy and chemotherapy in patients with locally advanced and metastatic bladder urothelial carcinoma. Immunotherapy was reported to be superior to chemotherapy in terms of high grade adverse events, patients’ compliance, and quality of life in both first-line and second-line therapy for those patients (56). Furthermore, most current guidelines recommend avelumab as first line maintenance therapy after platinum-based chemotherapy as the new standard for patients with locally advanced or metastatic urothelial carcinoma. In a phase III study of 700 patients with advanced or metastatic urothelial carcinoma who did not have disease progression following first-line chemotherapy, avelumab significantly improved survival compared to the patients who only had supportive care (57). The 1-year survival was 71.3% and 60.4% for the avelumab group and supportive care group, respectively. Thus, patients who respond to the induction chemotherapy can be offered avelumab first-line maintenance therapy until disease progression or unacceptable adverse events (58, 59).

However, in contrast to studies using immunotherapy for locally advanced bladder MIBC, there are still controversies about the role of biomarkers in patients with metastatic bladder cancer, as patients with low PD-L1 expression may also have similar survival after immunotherapy compared to those with higher expression (60). We postulate that the difference in tumor response may have been related to the tumor microenvironment of the distant metastases, which have been reported to differ from the primary sites in different tumors (61–64). Biopsies of the primary tumor and their metastases demonstrated a discordance between PD-L1 expression and a tumor microenvironment which is less responsive to immunotherapy (61, 62). However, more investigations need to be done as most clinicians assume that the tumor microenvironment is similar between the primary tumor and the distant metastatic sites. Thus, biopsy of the distant metastases is frequently not performed, and treatment decision of stage four disease relies on the biomarkers of the primary site (65).

### Effectiveness of radiotherapy to enhance tumor killing by ICI

*In vivo* experiments have demonstrated the effectiveness of high-dose radiotherapy to improve survival among animals who were inoculated with bladder cancer cells. Compared to placebo, mice who developed bladder cancer had significantly improved survival when treated with radiotherapy alone, ICI alone, and ICI combined with radiotherapy. The group who received the combined treatment had the best survival (66). Part of the survival improvement was due to the abscopal effect of radiotherapy as the chemokine C-X-C motif ligand 9 (CXCL) was upregulated of the combined treatment group, leading to an increase of CD8+ T cells and natural killer (NK) cells in the tumor (66). Timing of the radiation before, during, or after ICI did not affect survival for those receiving radiotherapy and ICI (67). Thus adding radiotherapy to immunotherapy was the key for survival benefit. In patients with low PD-L1 expression (<1%), radiotherapy upfront may be advantageous as it upregulates PD-L1 expression of tumor cells, thus making them more sensitive to ICI (32).

The induction of PD-L1 formation following radiotherapy is not specific to bladder cancer as it has been reported among many tumors with different histology both in the laboratory and in clinical studies.

Using immunofluorescence and three-dimensional structured illumination spectroscopy, Permata et al. (68) demonstrated a substantial increase in PD-L1 expression following irradiation of osteosarcoma cells lines with various doses of carbon-ion and X-ray irradiation. The increase in PD-L1 expression was greater with carbon-ion suggesting that high linear-energy transfer particles irradiation may be more effective compared to photons. Prostate cancer allografts also experienced delayed growth and an increase in PD-L1 expression following three fractions of 5 Gy seven days after irradiation (69). In another study using immunoPET/CT imaging by Zr-89-labeled anti-PD-L1 monoclonal antibody, Kikuchi et al. (70), reported a significant elevation of PD-L1 of head and neck and melanoma cancer implanted in mice after radiotherapy with two fractions of 2 Gy times 4 or 10 fractions. The increase in PD-L1 expression is dose-dependent among tumors which have little baseline PD-L1 expression such as esophageal adenocarcinoma (71). These *in vitro* and *in vivo* experiments supported the role of irradiation in the upregulation of PD-L1 expression.

Clinical studies also corroborate the impact of radiotherapy on the expression of PD-L1 in tumor cells. Even among tumors that do not express PD-L1 at diagnosis, radiotherapy administration may turn them PD-L1 positive. Among 46 patients with extremities sarcoma who were PD-L1 negative on initial biopsy, following preoperative radiotherapy to a dose ranging from 45 Gy to 50 Gy, 10.6% became positive after irradiation (72). In other studies preoperative radiotherapy or chemoradiation enhanced PD-L1 expression. Boustani et al. (73) reported the PD-L1 expression in 74 patients who underwent preoperative radiotherapy or chemoradiation for locally advanced rectal cancer. PD-L1 expression was 15% and 50% before and after irradiation, respectively. Corresponding figures for 75 patients who underwent chemoradiation for cervical cancer were 5% and 52%, respectively (74). In patients with non-small cell lung cancer, not only PD-L1 expression in the biopsy specimen increased from 1% to 48% after chemoradiation, but there was also a significant increase in PD-L1 expression in circulating tumor cells (CTC) during treatment, suggesting a natural response of tumor cells to escape the immune response induced by radiotherapy (75, 76).

Upregulation of PD-L1 in tumor cells and in the tumor microenvironment by radiotherapy is a complex mechanism and thought to be through four pathways: Interferon γ signaling, epidermal growth factor receptor pathway, DNA damage signaling pathway, and cGAS-STING pathway (77). Increase in PD-L1 expression allows the tumor cells to escape killing by CD-8+ T cells, which are attracted to the tumor microenvironment after radiation through binding of T cells program death 1 (PD-1) receptor (78). Thus, clinicians can formulate a policy to combine immunotherapy with radiotherapy to improve local control and survival not only for bladder cancer but also other tumor types such as non-malanoma skin cancer (79).

Preliminary studies suggest that the combination of immunotherapy and radiotherapy may be feasible with acceptable toxicity. Among 32 patients with clinical stage T2–4aN0M0 who were not eligible for surgery or declined cystectomy, TURBT was performed followed by immunotherapy with durvalumab and tremelimumab every four weeks for three doses. Radiotherapy was initiated two weeks after immunotherapy to a total dose of 64 Gy to 66 Gy and 46 Gy to the bladder and pelvic lymph nodes, respectively. 26 patients (81%) achieved a biopsy proven CR after treatment which was significantly higher than the ones reported after neoadjuvant immunotherapy ranging from 14% to 46% (**Table 1**). Grade 3–4 toxicity was 34% (80). Another study corroborated the efficacy of pembrolizumab as a second-line treatment for locally advanced bladder cancer in combination with radiotherapy. Among 12 patients treated with curative intent, median survival was 27.7 months. There was no difference in grade 3–4 toxicity compared to a group of patients who was treated with pembrolizumab alone (81). Thus, given the synergy between immunotherapy and radiotherapy, further prospective studies are needed to select patients who are most likely to benefit from the combined treatment while minimizing treatment toxicity.

### Efficacy of immunotherapy among older cancer patients with bladder cancer

Preliminary studies suggest that ICI, and in particular pembrolizumab, are well tolerated and effective among older patients with bladder cancer and a poor performance status. Among advanced bladder cancer patients who were ineligible for cisplatin due to their age and poor performance status (Eastern Cooperative Oncology Group performance status score 2), pembrolizumab was administered as first-line therapy every three weeks until disease progression, intolerable toxicity, or 24 months of therapy. There was no difference in response rate, survival, and toxicity between patients aged 65 or older (n=302) and 75 or older (n=179) (82). Another study using real-world data corroborated the efficacy and safety of pembrolizumab for older patients with advanced bladder cancer who progressed after chemotherapy. There was no difference in survival or grade 3–4 toxicity between patients less than 75-year-old (n=215) or 75-year-old or older (n=215) (83). Other ICI are also well tolerated in older patients with urothelial carcinoma (84). These studies emphasized the safety profile of ICI for the treatment of other solid tumors in older patients (85–88).

### Image-guided radiotherapy for the treatment of locally advanced MIBC.

Radiotherapy has been an effective treatment for locally advanced bladder cancer either alone or combined with chemotherapy. However, radiotherapy planning is difficult due to the distensibility of the bladder, leading to potential marginal miss and/or serious toxicity from excessive irradiation of the normal organs surrounding the target (89). It is also very difficult to deliver a high dose to the gross tumor volume (GTV) as it is not well delineated on the planning CT scan. An ideal radiotherapy technique would deliver a very high dose to the GTV while minimizing dose to the organs at risk (OAR) to decrease the risk of complications.

Fiducial markers are critical to delineate the bladder GTV for accurate radiotherapy delivery (90). Two fiducial markers, gold seeds and Lipiodol, are available to outline the GTV. Even though they are equally effective, the advantage of Lipiodol is the relative technical ease for injection and the absence of risk linked to seed migration after its insertion (90). Thus, for practical purpose, Lipiodol may be the preferred fiducial method for clinical studies involving multiple institutions (91).

Following TURBT, a soluble iodinated radiocontrast agent, Lipiodol, is injected through flexible cystoscopy into the bladder submucosa circumferentially 2–3 mm from the margin of resection or the visible GTV. The contrast agent remains visible during the conventional seven-weeks course of radiotherapy. Many studies have investigated the safety and visibility of Lipiodol on planning CT scan and cone beam CT scan during radiotherapy (92–95). As an illustration, Nakamura et al. (95) emphasized the feasibility of partial bladder tumor boost with Lipiodol toward the end of the treatment with IGRT, which decreased the risk of long-term cystitis while allowing long-term local control.

Advancements in radiotherapy techniques like intensity-modulated radiotherapy (IMRT) and image-guided radiotherapy (IGRT) have allowed clinicians to accurately deliver a high tumor dose while minimizing OAR’s dose, thus improving local control and reducing serious complications in older patients with locally advanced MIBC (96).

A review of the literature on locally advanced MIBC treated with IGRT alone or combined with chemotherapy corroborates that the normal organs sparing of this technique translates into improved tolerance to radiotherapy for older cancer patients. Acute grade 3–4 toxicity ranged from 2.3% to 30.3%. Long-term toxicity was low and ranged from 0 to 11.5% (97–104). Local control ranges from 56% to 78% depending on the length of the follow-up. However, there was no consensus on the dose and target volume delineation. For frail and old cancer patients, an ultra-weekly hypofractionation of 6 Gy times 6 to the bladder was well tolerated (99, 102, 104). Chemotherapy is omitted for those patients due to their frailty status. Other studies used an integrated boost technique to deliver a higher dose to the GTV concurrently with chemotherapy to minimize toxicity for patients with a better performance status, as the pelvic lymph nodes and bladder received a lower dose (90, 91). Overall, hypofractionated radiotherapy was well tolerated and might be best suited for older cancer patients to decrease their need for transportation. **Table 2** summarizes the studies on IGRT for locally advanced MIBC.

**Table 2:** Image-guided radiotherapy for locally advanced muscle invasive bladder cancer.

Study	Patient	Radiation	dose		Chemotherapy	Local control	Complications	
	No	Pelvis	Bladder	GTV			Acute	Late
Murthy et al (97)	44	55 Gy	64 Gy	68 Gy	Yes	78% (3 year)	11% gr 3	4% gr 3
Kang et al (98)	26	45 Gy	45 Gy	62.5 Gy	Yes	86% (2 year)	3.8% gr 4	11.5% gr. 3
Huddart et al (99)	33		36 Gy		No	71.7% (1 year)	30.3% gr 3-4	11.5% gr 2-4
Navarro et al (100)	117	NS	55-60Gy		Yes	56% (5 year)	4% gr 3-4	4% gr 3-4
Remonde et al (101)	300	NS	59.4 Gy		Yes	71.7 (5 year)	NS	NS
Zygogianni et al (102)	43		36 Gy		No	NS	2.3%	O%
Hsieh et al (103)	10	NS	57.6 Gy		Yes	83.3% (2 year)	10% gr 3	NS
Hafeez et al (104)	55		36 Gy		No	83% (2 year)	22% gr 3	4.3% gr 3

No, number; NS, non specified; gr, grade; Gy, gray; GTV, gross tumor volume.

### Evaluation of frailty in older patients with locally advanced MIBC

Evaluation of frailty in older patients (defined as 65 years old or above) with locally advanced MIIBC is crucial before enrolling them in any protocol, given its impact on treatment outcomes. Frailty is defined as a state of increased vulnerability resulting from aging associated decline in reserve and function across multiple physiologic systems (105). As a result, the body’s ability to deal with stress is altered. In frail cancer patients, there is an increase mortality risk with surgery and chemotherapy (10, 106). There are several questionnaires to assess frailty in older patient, with the G-8 questionnaire being practical to implement in clinical trials due to its simplicity (107). Patients with a score of 15 or above are defined as fit while those with a score of 14 or less undergo a complete geriatric assessment with the comprehensive geriatric assessment (CGA) survey (108). We propose a protocol using patient fitness and biomarkers to stratify treatment of older patients with locally advanced MIBC who are not candidates for cisplatin chemotherapy and surgery.

### Proposed IGRG algorithm for older patients with locally advanced MIBC

All tumor biopsy specimen should undergo next generation sequencing (NGS), if feasible, which includes PD-L1 and TMB status.

Patients with PD-L1 with 1% or more and/or TMB equal or more than 13 mut/MB should undergo immunotherapy as the first-line of treatment for four cycles before radiotherapy as they are likely to respond to ICI. Four cycles of immunotherapy are proposed instead of the two to three cycles reported for neoadjuvant immunotherapy, with the hypothesis that it may further improve the pCR rate. Notably, the pCR was higher for three cycles compared to two cycles with single agent ICI (**Table 1**). Thus, adding one cycle, similar to the protocol for patients who underwent neoadjuvant immunotherapy for locally advanced squamous cell carcinoma of the skin, may be beneficial (99, 109). Gross et al. (109) reported a pCR rate of 51% for those patients.

For frail patients, we propose a regimen of 6 Gy weekly for six weeks to the bladder with IGRT two weeks following immunotherapy as this regimen is well tolerated for older patients. We believe that sequential treatment works best to minimize treatment toxicity, as significant toxicity was reported with concurrent immunotherapy and weekly radiotherapy for bladder cancer. In a phase I study of five patients with bladder cancer who underwent concurrent immunotherapy and concurrent immunotherapy, four patients developed grade 3–4 toxicity (110).

Fit patients should receive a hypofractionated regimen, which includes treating the pelvic lymph nodes, bladder, and GTV to a total dose of 44 Gy in 2.2 Gy/fraction, 50 Gy in 2.5 Gy/fraction and 55 Gy in 2.75 Gy/fraction, respectively, with the simultaneous integrated boost technique to minimize treatment toxicity. Corresponding biologic equivalent dose (BED) would be 45.22, 62.5, and 70.1 Gy, respectively.

For patients with PD-L1 less than 1% and TMB less than 13 mut/MB, radiotherapy should be administered first to induce upregulation of PD-L1, followed by four cycles of immunotherapy. The radiotherapy dose and fractionation are identical for frail and fit patients.

External beam pelvic irradiation should be performed with IMRT and IGRT to minimize complication rates. The GTV should be outlined with Lipiodol or another fiducial marker depending on the institution’s expertise. Patients who respond to the combination of radiotherapy and immunotherapy may be offered avelumab maintenance therapy until disease progression or unacceptable adverse events occur, at the discretion of the investigator.

Conclusions based on prospectively collected data would improve the design of future clinical trials targeting older patients treated with immunotherapy and radiotherapy for bladder cancer.

**Figure 1:**
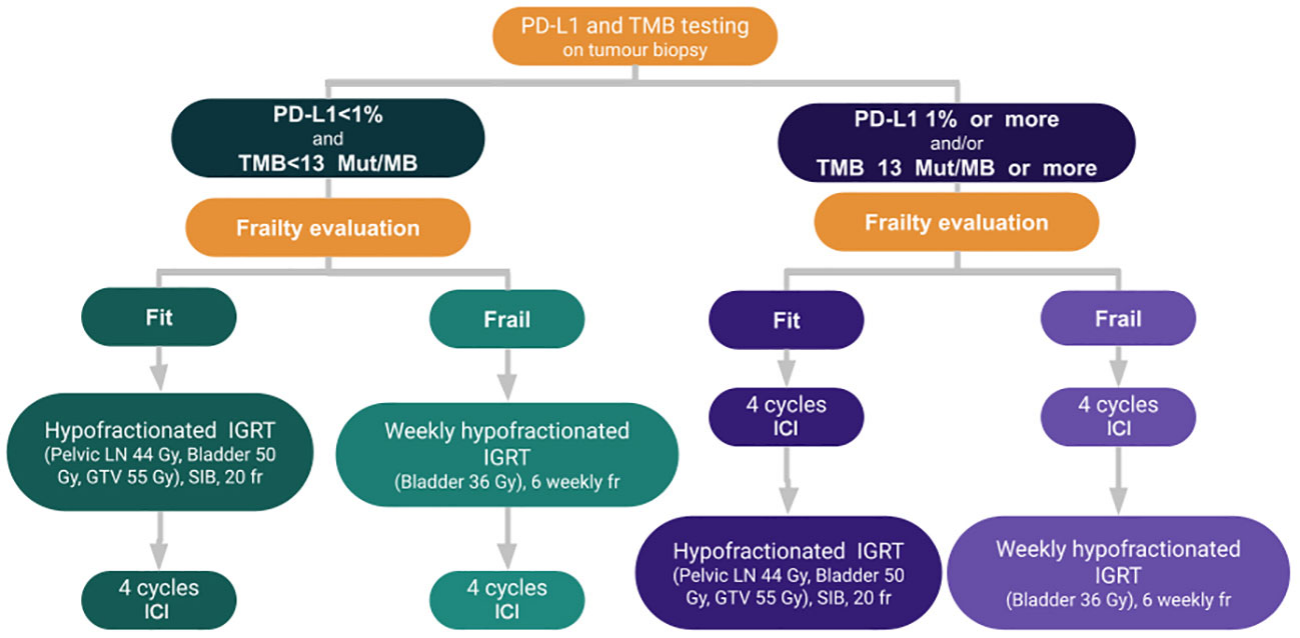
Treatment algorithm for muscle invasive bladder cancer.

**Figure 1** summarizes the proposed algorithm.

The IGRG is committed to conducting such studies when funding becomes available, leveraging its network of cancer institutions worldwide (n=1282) and diverse patient population (111, 112).

## Conclusion

The combination of radiotherapy and immunotherapy may be beneficial for older patients with locally advanced MIBC who are not eligible for cisplatin chemotherapy and are not candidates or decline cystectomy. Prospective studies should be conducted to verify this hypothesis.

## Data availability statement

The original contributions presented in the study are included in the article/supplementary material. Further inquiries can be directed to the corresponding author/s.

## Author contributions

NN: Formal analysis, Writing – original draft, Writing – review & editing. UK: Conceptualization, Formal analysis, Writing – original draft, Writing – review & editing. BP: Conceptualization, Formal analysis, Writing – original draft, Writing – review & editing. M-EC: Conceptualization, Formal analysis, Writing – original draft, Writing – review & editing. VV-H: Conceptualization, Formal analysis, Writing – original draft, Writing – review & editing. OG: Conceptualization, Formal analysis, Writing – original draft, Writing – review & editing. MA: Conceptualization, Writing – original draft, Writing – review & editing. MM: Conceptualization, Formal analysis, Writing – original draft, Writing – review & editing. SJ: Conceptualization, Formal analysis, Writing – original draft, Writing – review & editing. HG: Conceptualization, Formal analysis, Writing – original draft. LK: Conceptualization, Formal analysis, Writing – original draft, Writing – review & editing. FD: Conceptualization, Formal analysis, Writing – original draft, Writing – review & editing. VM: Conceptualization, Formal analysis, Writing – original draft, Writing – review & editing. AMa: Conceptualization, Formal analysis, Writing – original draft, Writing – review & editing. GT: Conceptualization, Formal analysis, Writing – original draft, Writing – review & editing. ZD: Conceptualization, Formal analysis, Writing – original draft, Writing – review & editing. GL: Conceptualization, Writing – original draft, Writing – review & editing. SeB: Conceptualization, Formal analysis, Writing – original draft, Writing – review & editing. SaB: Conceptualization, Formal analysis, Writing – original draft, Writing – review & editing. EN: Conceptualization, Formal analysis, Writing – original draft, Writing – review & editing. EL: Conceptualization, Formal analysis, Writing – original draft, Writing – review & editing. AMo: Conceptualization, Formal analysis, Writing – original draft, Writing – review & editing.

## References

[1] Lin W Pan X Zhang C Ye B Song J. Impact of age on diagnosis of bladder cancer on survival: A surveillance, epidemiology, and end results-based study 2004-2015. Cancer Control. (2023) 30:1–4. doi: 10.1177/10732748231152322 PMC9903028 36662642

[2] Milowsky MI Rumble RB Booth CM Gilligan T Eapen LJ Hauke RJ. Guideline on muscle-invasive and metastatic bladder cancer (Eurpean Association of Urology guideline): American Society of Clinical Oncology clinical practice guideline endorsements. J Clin Oncol. (2016) 34:1945–52. doi: 10.1200/JCO.2015.65.9797 27001593

[3] Skinner EC. Treatment of muscle invasive bladder cancer in older patients. Am Soc Clin Educ Book. (2016) 36:e228–3. doi: 10.14694/EDBK_158974 27249728

[4] Leminski A Kaczmarek K Golab A Kotfis K Skonieczna-Zydecka K Stozewski M. Muscle invasive bladder cancer: a retrospective observational comparative study. Clin Interv Aging. (2022) 17:255–63. 10.2147/CIA.S352890 PMC8922233 35299721

[5] Stamatakos PV Moschotzopoulos D Glykas I Fragkoulis C Kostakopoulos N Papapoulos G. Outcomes of radical cystectomy in pT4 bladder cancer frail patients: A high volume single center study. JFSF. (2022) 7:147–50. doi: 10.22540/JFSF-07-147 PMC9433942 36119554

[6] Choueiri TK Jacobus S Bellmunt J Qu A Appleman LJ Tretter C. Neoadjuvant dose-dense methotrexate, vinblastine, doxorubicin, and cisplatin with pegfilgrastim support in muscle-invasive urothelial cancer: pathologic, radiologic, and biomarkers correlates. J Clin Oncol. (2014) 32:1889–94. doi: 10.1200/JCO.2013.52.4785 PMC7057274 24821883

[7] de Haar-Hollerman A van Hoogstraten LMC Hulshof MCCM Tascilar M Bruck K Meijer RP. Chemoradiation for muscle invasive bladder cancer using 5- fluorouracil versus capecitabine: A nationwide cohort study. Radiother Oncol. (2023) 183:109584. doi: 10.1016/j.radonc.2023.109584 36863459

[8] Mallappallil M Friedman EA Delano BG McFarlane SI Salifu MO. Chronic kidney failure in the elderly. Clin Pract. (2014) 11:525–35. doi: 10.2217/cpr.14.46 PMC4291282 25589951

[9] Rosansky SJ Schell J Shega J Scherer J Jacobs L Couchoud C. Treatment decisions for older adults with advanced chronic kidney disease. BMC Nephrol. (2017) 18:200. doi: 10.1186/s12882-017-0617-3 28629462 PMC5477347

[10] Ho Y Tang W Chen S Lee S Chen J Hung Y. Association of frailty and chemotherapy-related adverse outcomes in geriatric patients with cáncer: a pilot observational study in Taiwan. Aging. (2021) 13:24192–204. doi: 10.18632/aging.v13i21 PMC8610137 34747717

[11] Chou W Lai C Hung C Hsueh S Yeh K Lu C. Clinical significance of frailty on treatment outcome in non-geriatric patients with head and neck cáncer and esophageal cáncer undergoing curative intent concurrent chemoradiotherapy. Cancer Control. (2022) 29:1–10. doi: 10.1177/10732748211045276 PMC8744172 34994207

[12] James ND Hussain SA Hall E Jenkins P Tremlett J Rawlings C. Radiotherapy with or without chemotherapy for muscle invasive bladder cáncer. N Engl J Med. (2012) 366:1477–88. doi: 10.1056/NEJMoa1106106 22512481

[13] Rompre-Brodeur A Shinde-Jadhav S Ayoub M Piccirillo CA Seuntjens J Brimo F. PD-1/PD-L1 check point inhibition with radiation in bladder cáncer: in *situ* and abscopal effects. Mol Cancer Thera. (2020) 19:211–20. doi: 10.1097/01.JU.0000556624.17353.4f 31534011

[14] Balar AV Kamat AM Kulkarni GS Uchio EM Boormans JL Roumiguie W. Pembrolizumab monotherapy for the treatment of high risk non muscle invasive bladder cáncer unresponsive to BCG (Keynote-057): an open label, single arm, multicentre, phase 2 study. Lancet Oncol. (2021) 22:919–30. doi: 10.1016/S1470-2045(21)00147-9 34051177

[15] Black PC Tangen C Singh P McConkey DJ Lucia S Lawrance WT. Phase II trial of atezolizumab in BCG-unresponsive non-muscle invasive bladder cáncer: SWOG S1605 (NCT02844816). J Clin Oncol. (2021) 39:4541. doi: 10.1200/JCO.2021.39.15_suppl.4541

[16] Peyrottes A Ouzaid I Califano G Hermieu J Xylinas E. Neoadjuvant immunotherapy for bladder cáncer. Medicina. (2021) 57:769. doi: 10.3390/medicina57080769 34440975 PMC8398505

[17] Nguyen NP Baumert BG Oboite E Motta M Appalanaido GK Arenas M. Immunotherapy and radiotherapy for older cáncer patients: proposed paradigm by the International Geriatric Radiotherapy Group. Gerontology. (2021) 67:379–85. doi: 10.1159/000514451 PMC8089416 33784693

[18] Xu H Clemenceau JR Park S Choi J Lee SH Hwang TH. Spatial heterogeneity and organization of tumor mutation burden with immune infiltrates within tumors based on whole slide images correlated with patient survival in bladder cáncer. J Pathol Inf. (2022) 13:100105. doi: 10.1016/j.jpi.2022.100105 PMC9577053 36268064

[19] Wu Z Wang M Liu Q Zhu K Chen L Guo H. Identification of gene expression profiles and immune cells infiftration signatures between low and high tumor mutation burden groups in bladder cáncer. Int J Med Sci. (2020) 17:89–96. doi: 10.7150/ijms.39056 31929742 PMC6945555

[20] Voutsadakis IA. Urothelial bladder carcinoma with a high tumor mutation burden have a better prognosis and targetable molecular defects beyond immunotherapy. Curr Oncol. (2022) 19:1390–407. doi: 10.3390/curroncol29030117 PMC8947463 35323317

[21] Lu J Zhu Y Ji A Zhang Q Liao G. Mining TCGA database for tumor mutation burden and their clinical significance in bladder cáncer. Biosci Rep. (2020) 40:1–12. doi: 10.1042/BSR20193421 PMC7178217 32239176

[22] Pfannstiel C Strissel PL Chiapinelli KB Sikic D Wach S Wirtz RM. The tumor microimmune environment drives a prognostic relevance that correlates with bladder cáncer subtypes. Cancer Immunol Res. (2019) 7:923–38. doi: 10.1158/2326-6066.CIR-18-0758 30988029

[23] Zhang Y Wang Y Wang J Zhang K. The immune cell infiltration and patterns and characterization score in bladder cáncer to identify the diagnosis. Front Genet. (2022) 13:852708. doi: 10.3389/fgene.2022.852708 35801082 PMC9255635

[24] Wang Z Zhu L Stebbing J Wang Z Peng L. Identification of an immune gene-associated prognostic signature in patients with bladder cáncer. Cancer Gene Ther. (2022) 29:494–504. doi: 10.1038/s41417-022-00438-5 35169299

[25] Huang T Chen X Zhang H Liang Y Li L Wei H. Prognostic role of tumor mutation burden in cáncer patients treated with immune check point inhibitors. Front Oncol. (2021) 11:706652. doi: 10.3389/fonc.2021.706652 34395281 PMC8358612

[26] Zheng M. Tumor mutation for predicting immune check point blockade response: the more the better. J Imunother Cancer. (2022) 10:e003087. doi: 10.1136/jitc-2021-003087 PMC8804687 35101940

[27] Tawahara T Ishiguro Y Ohtake S Kato I Ito Y Ito H. PD-1 and PD-L1 are more highly expressed in high grade bladder cáncer than in low grade cases: PD-L1 might function as a mediator of stage progression in bladder cáncer. BMC Urol. (2018) 18:97. doi: 10.1186/s12894-018-0400-1 30400941 PMC6219206

[28] Toren P Brisson H Simonyan D Hovington H Lacombe L Bergeron A. Androgen receptor and immune cell PD-L1 expression in bladder tumors predict disease recurrence and survival. World J Urol. (2021) 39:1549–58. doi: 10.1007/s00345-020-03358-x 32676741

[29] Chen L Han X. Anti-PD1/PD-L1 therapy of human cáncer. J Clin Invest. (2015) 9:3384–91. doi: 10.1172/JCI80011 PMC4588282 26325035

[30] Hashizume A Umemoto S Yokose T Nakamura Y Yoshihara M Shoji K. Enhanced expression of PD-L1 in non-muscle-invasive bladder cáncer after treatment with Bacillus Calmette-Guerin. Oncotarget. (2018) 9:34066–78. doi: 10.18632/oncotarget.v9i75 PMC6183350 30344922

[31] Zavalishina L Tsimafeyeu I Povilaitite P Raskin G Andreeva Y Petrov A. RUSSCO-RSP comparative study of immunohistochemistry diagnostic assays for PD-L1 expression in urothelial bladder cáncer. Virchows Archiv. (2018) 473:719–24. doi: 10.1007/s00428-018-2453-7 30209552

[32] Wu T Chen W Chang Y Lin W Chen M. The role of PD-L1 in the radiation response clinical outcome for bladder cáncer. Sci Rep. (2016) 6:1974. doi: 10.1038/srep19740 PMC4726250 26804478

[33] Pichler R Heidegger I Fritz J Danzl M Sprung S Zelger B. PD-L1 expression in bladder cáncer and metástasis and its influence on oncologic outcome after cystectomy. Oncotarget. (2017) 8:66849–64. doi: 10.18632/oncotarget.v8i40 PMC5620140 28978000

[34] Inman BA Longo TA Ramalingam S Harrison MR. Atezolizumab: A PD-L1 blocking antibody for bladder cáncer. Clin Cancer Res. (2017) 23:1886–90. doi: 10.1158/1078-0432.CCR-16-1417 27903674

[35] Rouanne M Radulescu C Adam J Allory Y. PD-L1 testing in urothelial bladder cáncer: essentials of clinical practice. World J Urol. (2021) 39:1345–55. doi: 10.1007/s00345-020-03498-0 33141317

[36] Liu C Liu Z Jin K Zeng H Shao F Chang Y. Integrative tumor burden with CD39 and PD-L1 for the prediction of response to PD-L1 blockade and adjuvant chemotherapy in muscle-invasive bladder cáncer patients. Br J Cancer. (2022) 127:1718–25. doi: 10.1038/s41416-022-01943-y PMC9596489 35999267

[37] Black PC Tangen C Singh P McConkey DJ Lucia S Lowrance WT. Phase II trial of atezolumab in BCG-unresponsive non-muscle invasive bladder cáncer: SWOG S1605. J Clin Oncol. (2021) 39:4541. doi: 10.1200/JCO.2021.39.15_suppl.4541

[38] Vandeveer AJ Fallon JK Tughe R Sabvezari H Schlom J Greiner JW. Systemic therapy of non-muscle invasive mouse bladder cáncer with avelumab, an anti-PD-L1 immune checkpoint inhibitor. Cancer Immunol Res. (2016) 4:452–62. doi: 10.1158/2326-6066.CIR-15-0176 PMC4881865 26921031

[39] Petrelli F Coinu A Cabiddu M Ghilardi M Vavassori I Barni S. Correlation of pathologic complete response with survival after neoadjuvant chemotherapy in bladder cáncer treated with cystectomy: A meta-analysis. Eur Urol. (2014) 65:350–7. doi: 10.1016/j.eururo.2013.06.049 23849998

[40] Hamid ARAH Ridwan FR Parikesit D Widia F Mochtar CA Umbas R. Meta- analysis of neoadjuvant chemotherapy compared to radical cystectomy alone in improving overall survival of muscle-invasive bladder cáncer patients. BMC Urol. (2020) 20:158. doi: 10.1186/s12894-020-00733-z 33054762 PMC7557048

[41] Bandini M Ross JS Raggi D Gallina A Colecchia M Luciano R. Predicting the complete pathologic response after neoadjuvant pembrolizumab in muscle invasive bladder cáncer. J Natl Cancer Inst. (2021) 113:48–53. doi: 10.1093/jnci/djaa076 32516377 PMC7781448

[42] Powles T Kockx M Rodriguez-Vida A Duran I Crabb SJ van der Heijden MS. Clinical efficacy and biomarker analysis of neoadjuvant atezolizumab in operable urothelial carcinoma in the ABACUS trial. Nat Med. (2019) 25:1706–14. doi: 10.1038/s41591-019-0628-7 31686036

[43] Basile G Bandini M Gibb EA Ross JS Raggi D Marandino L. Neoadjuvant pembrolizumab and radical cystectomy in patients with muscle invasive bladder cáncer: 3-year-median follow-up of the update of PURE-01 trial. Clin Cancer Res. (2022) 28:5108–14. doi: 10.1158/1078-0432.CCR-22-2158 36190522

[44] Hu J Chen J Ou Z Chen H Liu Z Chen M. Neoadjuvant immunotherapy, chemotherapy, and combination therapy in muscle-invasive bladder cáncer: a multi- center real-world retrospective study. Cells Rep Med. (2022) 3:100785. doi: 10.1016/j.xcrm.2022.100785 PMC9729796 36265483

[45] Necchi A Raggi D Gallina A Madison R Colecchia M Luciano R. Updated results of PURE-01 with preliminary activity of neoadjuvant pembrolizumab in patients with muscle-invasive bladder carcinoma with variant histologies. Eur Urol. (2020) 77:439–46. doi: 10.1016/j.eururo.2019.10.026 31708296

[46] Li R Nocera L Rose KM Raggi D Naidu S Mercinelli C. Comparative effectiveness of neoadjuvant pembrolizumab versus cisplatin-based chemotherapy or upfront radical cystectomy in patients with muscle-invasive urothelial bladder cáncer. Eur Urol Oncol. (2024) 7. doi: 10.1016/j.euo.2023.12.008 38184473

[47] Grivas P Koshkin VS Chu X Cole S Jain RK Dreicer R. PrECOG PrE0807: A phase I feasibility trial of neoadjuvant nivolumab without and with Lirilumab in patients with muscle-invasive bladder cáncer ineligible for or refusing cisplatin-based neoadjuvant chemotherapy. Eur Urol Oncol. (2023). doi: 10.1016/j.euo.2023.11.022 38155060

[48] van Dijk N Gil-Jimenez A Silina K Hendriksen K Smith LA de Feijter JM. Preoperative ipilimumab plus nivolumab in locoregionally advanced urothelial cáncer: the NABUCCO trial. Nat Med. (2020) 26:1839–44. doi: 10.1038/s41591-020-1085-z 33046870

[49] Grassauer J Schmidt J Cowan A Gilbert SM Chakiryan MH. Downstaging and survival associated with neoadjuvant immunotherapy before radical cystectomy for muscle-invasive bladder cáncer. Eur Urol Oncol. (2024) 7:139–46. doi: 10.1016/j.euo.2023.06.005 37453853

[50] Galsky MD Arija JAA Bamias A Davis ID de Santis M Kikuchi E. Atezolizumab with or without chemotherapy in metastatic urothelial cáncer: a multicenter, randomised, placebo controlled trial. Lancet. (2020) 395:1547–57. doi: 10.1016/S0140-6736(20)30230-0 32416780

[51] Rosenberg JE Hoffman-Censits J Pawles T van den Heijden MS Balar AJ Necchi A. Atezolizumab in patients with locally advanced and metastatic urothelial cancercinoma who have progressed following treatment with platinum-based chemotherapy: a single arm multicenter, phase II trial. Lancet. (2016) 387:1909–20. doi: 10.1016/S0140-6736(16)00561-4 PMC5480242 26952546

[52] Powles T Duran I van der Heijden MS Loriot Y Vogelzang NJ De Georgi U. Atezolizumab versus chemotherapy in patients with platinum treated locally advanced or metastatic urothelial carcinoma: a multicentre, open-label, randomized trial. Lancet. (2018) 391:748–57. doi: 10.1016/S0140-6736(17)33297-X 29268948

[53] Powles T van der Heijden MS Castellano D Galsky MD Loriot Y Petrylak DP. Durvalumab alone and durvalumab plus tremelimumab versus chemotherapy in previously untreated patients with unresectable, locally advanced or metastatic urothelial carcinoma: a randomized, open-label, multicentre, phase 3 trial. Lancet Oncol. (2020) 21:1574–88. doi: 10.1016/S1470-2045(20)30541-6 32971005

[54] Fradet Y Bellmunt J Vaughn DJ Lee JL Fong L Vogeljang NJ. Randomized phase III Keynote-045 trial of pembrolizumab versus paclitaxel, docetaxel, or vinflunine in recurrent advanced urothelial cáncer: results of >2 years follow-up. Ann Oncol. (2019) 30:970–6. doi: 10.1093/annonc/mdz127 PMC6594457 31050707

[55] Vuky J Balar AV Castellano D O’Donnell PH Grivas P Bellmunt J. Long- term outcomes in KEYNOTE-052: phase II study investigating first-line pembrolizumab in cisplatin ineligible patients with locally advanced or metastatic urothelial cáncer. J Clin Oncol. (2020) 38:2658–66. doi: 10.1200/JCO.19.01213 32552471

[56] Maisch P Hwang EC Kim K Naravan VM Bakker C Kunath F. Immmunotherapy for advanced or metastatic urothelial carcinoma. Cochrane Database Syst Rev. (2023) 10:CD013774. doi: 10.1002/14651858.CD013774.pub2 37811690 PMC10561349

[57] Powles T Park SH Voog E Cacerta C Valderrama BP Gurney H. Avelumab maintenance therapy for advanced or metastatic urothelial carcinoma. New Engl J Med. (2020) 383:1218–30. doi: 10.1056/NEJMoa2002788 32945632

[58] Mansinho A Cruz A Marconi L Pinto C Augusto I. Avelumab as first line maintenance treatment in locally advanced or metastatic urothelial carcinoma. Adv Ther. (2023) 40:4134–50. doi: 10.1007/s12325-023-02624-9 37608243

[59] Sridhar SS Powles T Climent Durant MA Park SH Massari F Thiery-Vuillemin A. Avelumab first line maintenance for advanced urothelial carcinoma: Analysis from Javelin Bladder 100 by duration of first line chemotherapy and interval before maintenance. Eur Urol. (2023) 85. 10.1016/j.eururo.2023.08.001 37714742

[60] Aggen DH Drake CG. Biomarkers for immunotherapy in bladder cáncer: A moving target. J Immunother Cancer. (2017) 5:94. doi: 10.1186/s40425-017-0299-1 29157296 PMC5697433

[61] Szekely B Bossuyt V Li X Wali VB Patwardhan GA Frederick C. Immunological difference between primary and metastatic breast cáncer. Ann Oncol. (2018) 29:2232–9. doi: 10.1093/annonc/mdy399 30203045

[62] Song SG Kim S Koh J Yim J Han B Kim JA. Comparative analysis of the tumor microenvironment of primary and brain metastases of non-small cell lung cáncer reveals organ-specific and EGFR mutation-dependent unique immune landscape. Cancer Immunol Immunother. (2021) 70:2035–48. doi: 10.1007/s00262-020-02840-0 PMC10992873 33420630

[63] Callea M Albiges L Gupta M Cheng S Genega EM Fay AP. Differential expression of PD-L1 between primary and metastatic sites in clear cell renal carcinoma. Cancer Immunol Res. (2015) 10:1158–64. doi: 10.1158/2326-6066.CIR-15-0043 PMC4596765 26014095

[64] Chen X Chen H He D Cheng Y Zhu Y Xiao M. Analysis of tumor microenvironment characteristics in bladder cancer. Front Immunol. (2021) 12:672158. doi: 10.3389/fimmu.2021.672158 33936117 PMC8082152

[65] Khasraw M Brogi E Seidman AD. The need to examine metastatic tissue at the time of progression of breast cáncer: Is re-biopsy a necessary or a luxury. Curr Oncol Rep. (2011) 13:17–25. doi: 10.1007/s11912-010-0137-9 21053108

[66] Rompre-Brodeur A Shinde-Jadhav S Ayoub M Piccirillo CA Seuntjens J Brimo F. PD-1/PD-L1 immune check point inhibition with radiation in bladder cáncer: *In situ* and abscopal effect. Mol Cancer Ther. (2020) 19:211–20. doi: 10.1158/1535-7163.MCT-19-0503 31534011

[67] Tholomier C Marcq G Shinde-Jadhav S Yahoub M Huang JM Kool R. Optimizing sequence of PD-L1 immune checkpoint inhibitors and radiation therapy in bladder Cáncer. Bladder cáncer. (2020) 6:295–302. doi: 10.3233/BLC-200315

[68] Permata TBM Sato H Gu W Kakoti S Uchihara Y Yoshimatsu Y. High linear energy transfer carbón-irradiation upregulates PD-L1 expression more significantly than X-rays in human osteosarcoma U2OS cells. J Radiat Res. (2021) 62:773–81. doi: 10.1093/jrr/rrab050 PMC8438258 34196706

[69] Philippou Y Sjoberg HT Murphy E Alyacoubi S Jones KI Gordon-Weeks AN. Impact of combining anti PD-L1 immunotherapy and radiotherapy on the tumor micro-environment in a murine prostate cáncer model. Br J Cancer. (2020) 123:1089–100. doi: 10.1038/s41416-020-0956-x PMC7525450 32641865

[70] Kikuchi M Clump DA Srivasta RM Sun L Zeng D Diaz-Perez. Preclinical immunoPET/CT imaging usinf Zr-89-labeled anti-PD-L1 antibody for assessing radiation-induced PD-L1 upregulation in head and neck cáncer and melanoma. Oncoimmunology. (2017) 19:e1329071. doi: 10.1080/2162402X.2017.1329071 PMC5543907 28811971

[71] Kelly R Zaidi A Smith M Omstead A Kosovec J Matsui D. The dynamic and transient immune microenvironment in locally advanced adenocarcinoma post chemoradiation. Ann Surg. (2018) 268:992–9. doi: 10.1097/SLA.0000000000002410 28806299

[72] Patel KR Martinez A Stahl JA Logan SJ Perricone AJ Ferris MJ. Increase in PD-L1 expression after pre-operative radiotherapy for soft tissue sarcoma. Oncoimmunology. (2018) 7:e1442168. doi: 10.1080/2162402X.2018.1442168 29900051 PMC5993497

[73] Boustani J Derangere V Bertaut A Adotevi O Morgand V Charron-Barra C. Radiotherapy scheme effect on PD-L1 expression for locally advanced rectal cancer. Cells. (2020) 9:2071. doi: 10.3390/cells9092071 32927784 PMC7563314

[74] Mori Y Sato H Kumazawa T Permata TBM Yoshimoto Y Murata K. Analysis of radiotherapy-induced alteration of CD8+ and PD-L1 expression in patients with uterine cervical carcinoma. Oncol Lett. (2021) 21:446. doi: 10.3892/ol 33868484 PMC8045163

[75] Yoneta K Kuwata T Kanayama M Mori M Kawanami T Yatera K. Alteration in tumoural PD-L1 expression and stromal CD8-positive tumor-infiltrating lymphocytes after concurrent chemo-radiotherapy for non-small cell lung cancer. Br J Cancer. (2019) 121:490–6. doi: 10.1038/s41416-019-0522-5 PMC6738061 31388183

[76] Wang Y Kim TH Fouladdel S Zhang Z Soni P Qin Y. PD-L1 expression in circulating tumor cells increases during radio(chemo)therapy and indicates poor prognosis in non-small cell lung cáncer. Sci Rep. (2018) 9:566. doi: 10.1038/s41598-018-36652-2 PMC6345864 30679441

[77] Sato H Okonogi N Yoshimoto Y Tamaki T Suzuki Y. Radiotherapy and PD-L1 expression. Cancer Chemother. (2019) 46:845–9. 31189801

[78] Wu M Huang Q Xie Y Wu X Ma H Zhang Y. Improvement of the anticancer efficacy of PD-1/PD-L1 blockade via combination therapy and PD-L1 regulation. J Hematol Oncol. (2022) 15:24. doi: 10.1186/s13045-022-01242-2 35279217 PMC8917703

[79] Nguyen NP Thariat J Gorobets O Vinh-Hung V Kim L Blanco SC. Immunotherapy and hypofractionated radiotherapy in older patients with locally advanced cutaneous squamous cell carcinoma of the head and neck: A proposed paradigm by the International Geriatric Radiotherapy Group. Cancers. (2023) 14:4981. doi: 10.3390/cancers15204981 PMC10605563 37894347

[80] del Muro XG Valderrama BP Medina A Cuellar MA Etxaniz O Sarrio RG. Phase II trial of durvalumab plus tremelimumab with concurrent radiotherapy in patients with localized muscle invasive bladder cáncer treated with a selective bladder preservation approach. J Clin Oncol. (2021) 39:4505. doi: 10.1200/JCO.2021.39.15_suppl.4505

[81] Sano T Aizawa R Ito K Nakamura K Ogata T Takeda M. Efficacy and tolerability of second line pembrolizumab with radiation therapy in advanced urothelial carcinoma. Anticancer Res. (2023) 43:2119–26. doi: 10.21873/anticanres.16373 37097696

[82] Grivas P Plimack ER Balar AV Castellano D O’Donnell PH Bellmunt J. Pembrolizumab as first-line therapy in cisplatin-ineligible advanced urothelial cancer (KEYNOTE-052): Outcomes in older patients by age and performance status. Eur Urol Oncol. (2020) 3:351–9. doi: 10.1016/j.euo.2020.02.009 PMC8246631 32423837

[83] Nishiyama N Kobayashi T Narita S Hikada Y Ito K Maruyama S. Efficacy and safety of pembrolizumab for older patients with chemoresistant urothelial carcinoma assessed using propensity score matching. J Geriat Oncol. (2022) 13:88–93. doi: 10.1016/j.jgo.2021.07.002 34238726

[84] Schulz B Rodler S Szabados B Graser A Buchner A Stief C. Safety, efficacy, and prognostic impact of checkpoint inhibitors in older patients with genitourinary cáncer. J Geriat Oncol. (2020) 11:1061–6. doi: 10.1016/j.jgo.2020.06.012 32565147

[85] Herin H Aspeslagh S Castanon E Dyevre V Marabelle A Varga A. Immunotherapy phase I trials in patients older than 70 years with advanced solid tumors. Eur J Cancer. (2018) 95:68–74. doi: 10.1016/j.ejca.2018.03.002 29635146

[86] Gomes F Lorigan P Woolley S Foden P Burns K Yorke J. A propective study of the safety of checkpoint inhibitors in older cáncer patients-the ELDERS study. Esmo Open. (2021) 6:1–6. doi: 10.1016/j.esmoop.2020.100042 PMC7844568 33516147

[87] Wu Q Wang Q Tang X Xu R Zhang L Chen X. Correlation between patients’ age and cáncer immunotherapy efficacy. Oncoimmunology. (2019) 8:e1568810. doi: 10.1080/2162402X.2018.1568810 30906662 PMC6422380

[88] Corbaux P Maillet D Boespflug A Locatelli-Sanchez M Periez-Muzet M Duruisseaux M. Older and younger patients treated with immune checkpoint inhibitors have similar outcomes in real life setting. Eur J Cancer. (2019) 1212:192–201. doi: 10.1016/j.ejca.2019.08.027 31590080

[89] Fokdal L Honore H Hoyer M Meldgaard P Fode K von der Maase H. Impact of changes in bladder and rectal filling volume on organ motion and dose distribution of the bladder in radiotherapy for urinary bladder cáncer. Int J Radiat Oncol Biol Phys. (2004) 59:436–44. doi: 10.1016/j.ijrobp.2003.10.039 15145160

[90] Della Bianca C Yorke ED Mechalakos JG Kollmeier MA. Comparison of setup by bony anatomy and by fiducial marker registration in image-guided radiotherapy for bladder cáncer. Int J Radiat Oncol Biol Phys. (2009) 75:S649.

[91] Nolan CP Forde EJ. A review of the use of fiducial markers for image-guided radiotherapy. Acta Oncol. (2016) 55:533–8. doi: 10.3109/0284186X.2015.1110250 26588169

[92] Baumgarten AS Emtage JB Wilder RB Biagioli MC Gupta S Spiess PE. Intravesical lipiodol injection technique for image-guided radiation therapy for bladder cáncer. Urology. (2014) 83:946–50. doi: 10.1016/j.urology.2013.09.058 24397940

[93] Chai X van Herk M van de Kamer JB Remeijer P Bex A Betgen A. Behavior of lipiodol markers during image-guided radiotherapy of bladder cáncer. Int J Radiat Oncol Biol Phys. (2010) 77:309–14. doi: 10.1016/j.ijrobp.2009.08.019 20137863

[94] Pos F Bex A Dees-Ribbers HM Betjen A van Herk M Remeijer P. Lipiodol injection for target volume delineation and image guidance during radiotherapy for bladder cáncer. Radiother Oncol. (2009) 93:364–7. doi: 10.1016/j.radonc.2009.09.003 19800703

[95] Nakamura R Kakuhara H Kikuchi K Segawa T Oikawa H Iwasaki K. Partial bladder boost using lipiodol marking during image-guided radiotherapy for bladder cáncer. Anticancer Res. (2018) 38:4827–31. doi: 10.21873/anticanres.12793 30061255

[96] Frick MA Ogunkeye J Oladipo ED Prado K Bagshaw HP. Radiotherapy vs cystectomy for treatment of muscle invasive bladder cáncer in very elderly patients. Int J Radiat Oncol Biol Phys. (2023) 117:2839. doi: 10.1016/j.ijrobp.2023.06.2498

[97] Murthy V Masodkar R Kalyani N Mahantshetty U Bakshi G Prakash G. Clinical outcomes with dose escalated adaptive radiation therapy for urinary bladder cáncer: a prospective study. Int J Radiat Oncol Biol Phys. (2016) 94:60–6. doi: 10.1016/j.ijrobp.2015.09.010 26547385

[98] Kang JJ Steinberg M Kupelian P Alexander S King CR. Whole versus partial bladder irradiation. Am J Clin Oncol. (2018) 41:107–14. doi: 10.1097/COC.0000000000000237 26535994

[99] Huddart R Hafeez S Lewis R McNair H Syndicus I Henry A. Clinical outcomes of a randomised trial of adaptive plan of the day treatment in patients receiving ultra-hypofractionated weekly radiation therapy for bladder cáncer. Int J Radiat Oncol Biol Phys. (2021) 110:411–24. doi: 10.1016/j.ijrobp.2020.11.068 PMC8114997 33316362

[100] Navarro-Domenech I Arulanantham S Liu ZA Tjong M Kong V Malkov V. Clinical and dosimetric outcomes of image-guided, dose-painted radiotherapy in muscle invasive bladder cáncer. Radiat Oncol. (2023) 18:154. doi: 10.1186/s13014-023-02338-w 37730609 PMC10512471

[101] Remonde D Saenz D Zhang J Kaushik D Rasmussen K Galvan E. Outcome of bladder preservation with combined modality treatment in the modern era of radiation therapy for muscle invasive bladder cáncer: A single tertiary cáncer center experience. Int J Radiat Oncol Biol Phys. (2020) 108:E918. doi: 10.1016/j.ijrobp.2020.07.557

[102] Zygogianni A Kouloulias V Armpilia C Antypas C Kantzou I Balafouta M. A weekly hypofractionated radiotherapeutic schedule for bladder carcinoma in elderly patients: local response, acute and late toxicity, dosimetric parameters and pain relief. JBUON. (2013) 18:407–12. 23818353

[103] Hsieh C Chung S Chan P Lai S Chang H Hsiao C. Intensity modulated radiotherapy for elderly bladder cáncer patients. Radiat Oncol. (2011) 6:75. doi: 10.1186/1748-717X-6-75 21679408 PMC3123577

[104] Hafeez S McDonald F Lalondrelle S McNair H Warren-Oseni K Jones K. Clinical outcomes of image guided adaptive hypofractionated weekly radiotherapy for bladder cáncer in patients unsuited for treatment. Int J Radiat Oncol Phys Biol. (2017) 98:115–22. doi: 10.1016/j.ijrobp.2017.02.223 PMC5392498 28586948

[105] Xue Q. The frailty síndrome: Definition and natural history. Clin Geriatr Med. (2011) 27:1–15. doi: 10.1016/j.cger.2010.08.009 21093718 PMC3028599

[106] Ethun CG Bilen MA Jani AB Maithel SK Ogan K Master VA. Frailty and cáncer: implication for oncology surgery, medical oncology, and radiation oncology. CA Cancer J Clin. (2017) 67:362–77. doi: 10.3322/caac.21406 28731537

[107] Takahashi M Takahashi M Komine K Yamada H Kasahara Y Chikamatsu S. The G8 screening tool enhances prognostic value to ECOG performance status in elderly cáncer patients: a retrospective, single institution study. PloS One. (2017) 12:e0179694. doi: 10.1371/journal.pone.0179694 28640844 PMC5480957

[108] Bellera CA Rainfray M Mathoulin-Pelissier S Mertens C Delva F Fonck A. Screening older cáncer patients: first evaluation of the G8 screening tool. Ann Oncol. (2012) 23:2166–72. doi: 10.1093/annonc/mdr587 22250183

[109] Gross ND Miller DM Khushalani NI Divi V Ruiz ES Lipson EJ. Neoadjuvant cimiplimab for stage II-IV cutaneous squamous cell carcinoma. N Engl J Med. (2022) 387:1557–68. doi: 10.1056/NEJMoa2118823 PMC9844515 36094839

[110] Tree AC Jones K Hafeez S Sharabiani MTA Harrington KJ Lalondrelle S. Dose-limiting urinary toxicity with pembrolizumab combined with weekly hypofractionated radiation therapy in bladder cáncer. Int J Radiat Oncol Biol Phys. (2018) 101:1168–71. doi: 10.1016/j.ijrobp.2018.04.070 30012528

[111] Popescu T Karlsson U Vinh-Hung V Trigo L Thariat J Vuong T. Challenges facing radiation oncologists in the management of older cancer patients: Consensus of the International Geriatric Radiotherapy Group. Cancers. (2019) 11:271. doi: 10.3390/cancers11030371 30884827 PMC6468336

[112] Nguyen NP Vinh-Hung V Baumert B Zamagni A Arenas M Motta M. Older cancer patients during the COVID-19 epidemic: practice proposal of the International Geriatric Radiotherapy Group. Cancers. (2020) 12:1287. doi: 10.3390/cancers12051287 32438703 PMC7281232

